# The Synergistic Effect of Exogenous Glutamine and Rifampicin Against *Mycobacterium* Persisters

**DOI:** 10.3389/fmicb.2018.01625

**Published:** 2018-07-20

**Authors:** Xue Huang, Xiangke Duan, Jiang Li, Jingjing Niu, Siqi Yuan, Xiaoyu Wang, Nzungize Lambert, Xue Li, Junqi Xu, Zhen Gong, Shuangquan Yan, Longxiang Xie, Jianping Xie

**Affiliations:** Institute of Modern Biopharmaceuticals, State Key Laboratory Breeding Base of Eco-Environment and Bio-Resource of the Three Gorges Area, Key Laboratory of Eco-environments in Three Gorges Reservoir Region, Ministry of Education, School of Life Sciences, Southwest University, Chongqing, China

**Keywords:** persister, glutamine, rifampicin, reactive oxygen species, *Mycobacteria*

## Abstract

Persisters, stochastic dormant variants of normal bacteria cell, represent a significant portion of the survivors upon exposure to antibiotics and other environmental stresses, which contributes substantially to high level antibiotics tolerance. Glutamine is a crucial component of the *Mycobacteria* nitrogen pool that is indispensable for survival upon stresses. To study whether a synergistic effect exists between glutamine and antibiotics against *Mycobacterial* persisters, the efficacy of rifampicin alone or together with exogenous glutamine upon *Mycobacterium smegmatis* mc^2^ 155 persisters was monitored. The result showed that glutamine decreases *M. smegmatis* tolerance to rifampicin upon starvation. The reactive oxygen species level of the strains treated with rifampicin and glutamine increased. The synergism of glutamine and rifampicin to kill persisters might derive from altering the oxidative phosphorylation and TCA cycle, as both evidenced by both ATP level increase and transcriptome change. Glutamine might represent a synergistic agent of rifampicin to kill *Mycobacteria* persisters.

## Introduction

Tuberculosis caused by *Mycobacterium tuberculosis* remains a serious global public health concern (Ginsberg and Spigelman, 2007; Gao et al., 2012), largely due to the emergence and widespread of multidrug-resistant strains and persisters recalcitrant to the current treatment regimen (Keren et al., 2004; Ginsberg and Spigelman, 2007). The pervasive alarmone ppGpp, a ubiquitous signal molecule produced by RelA, can trigger the antibiotic tolerance (Dahl et al., 2005; Traxler et al., 2008; Maisonneuve and Gerdes, 2014). Nitrogen is crucial for *Mycobacterium* cellular metabolites and virulence (Amon et al., 2009; Gouzy et al., 2014b; Williams et al., 2015). The degradation of glutamine, mediated by GlnA, can increase bacteria stress tolerance; similar effect was found for asparagine (Gouzy et al., 2013, 2014a). The glutamine/glutamate homeostasis is controlled via the glutamine synthesis capacity and supports bacterial growth under harsh conditions (Cowley et al., 2004). Glutamyl-tRNA mismatch can result in *M. tuberculosis* baseline tolerance against rifampicin (Javid et al., 2014). Upon glutamine starvation, numerous bacteria typically produce more ammonium carriers, high affinity assimilatory enzymes, and alternative enzymes for the nitrogen sources (Gouzy et al., 2014b). The regulon consists of ammonium transporters encoding genes *amtA*, *amtB*, *gdh*, *glnA*, and *gltBD*, which are crucial enzymes for *M. tuberculosis* survival upon stresses such as antibiotics exposure (Gouzy et al., 2014b). The importance of glutamine is well-recognized, nevertheless, its interaction with antibiotics against *M. tuberculosis* remains largely unknown.

In this study, *Mycobacterium smegmatis* was used to address whether glutamine can synergize with antibiotics against tolerance of *Mycobacterium* persister. The hallmark of persister is superb nutrition starvation survival ability. Amino acids played significant roles in persister tolerance (Magnusson et al., 2005; Traxler et al., 2008; Wu et al., 2010; Bernier et al., 2013; Lebeaux et al., 2014; Shan et al., 2015). Glutamine is essential for bacterial metabolism (di Mauro et al., 1969; Campbell et al., 2001; Keren et al., 2004, 2011; Gouzy et al., 2014b). Glutamine starvation can up-regulate the expression of stress responsive genes (Betts et al., 2002; Ye et al., 2009; Jessberger et al., 2013). Some amino acids, alone or with antibiotics, can inhibit the bacterial growth (Rowley, 1953; Amos and Cohen, 1954). Arginine can synergize with gentamicin against *Escherichia coli* (Lebeaux et al., 2014), and so does cysteine with isoniazid against *M. tuberculosis* (Vilcheze et al., 2017). To explore whether glutamine can synergize with rifampicin, the effect of rifampicin alone or with exogenous glutamine against persisters was compared. The addition of glutamine selectively synergized rifampicin against persisters. Both transcriptome and metabolome data revealed that glutamine and glutamate metabolism, as well as the contents of certain metabolites, cellular respiration, were altered, consistent with previous studies (Hu et al., 2003; Zhang et al., 2012). We hypothesized that the synergism between glutamine and rifampicin against *M. tuberculosis* provides a promising strategy to control *Mycobacterium* persister cells involved in ROS production.

## Materials and Methods

### Strains and Growth Conditions

Bacterial strains of *M. smegmatis* mc^2^ 155 were grown in 7H9 liquid medium (Difco) supplemented with 0.5% glycerol. According to Betts et al. (2002), with minor modification, the nutrition starvation culture (Albrethsen et al., 2013; Duan et al., 2016) was cultured at 37°C, 110 rpm, until OD_600_ = 0.8, and the culture was pelleted washed twice with 1× PBS, resuspended in 1× PBS, transferred to standing flasks or microplate, and incubated in the bottles (Shuniu) for 0, 48 or 72 h, until the treatment with antibiotics or amino acids. The final concentration of antibiotic is 100 μg/mL. The viability was determined by serial dilutions and plating on 7H10 agar.

### Drug Treatment of Cultures

For the drug treatment assay after nutrition starvation, the starved *M. smegmatis* culture was diluted 10 times in M9 medium without glucose at 0, 48, and 72 h time points, and 1 mL was aliquoted into each well of a 24-well plate, every sample with three replicates. Rifampicin was added to duplicate wells of cultures at a final concentration of 48.6–291.6 μM. Then concentration of 0–20 mM glutamine was added to *M. smegmatis* cultures. The MIC (μM) of rifampicin for *M. smegmatis* mc^2^ 155 was 4 μg/mL. The aqueous solutions of antibiotics and amino acids were filter sterilized by passing through a 0.22 μM syringe-fitted filter. Cultures were incubated with or without antibiotics at 37°C for 0–72 h, followed by serial diluting and plating on 7H10 agar to determine the bacterial viability.

### *In Vitro* Biofilm Formation and Treatment

Biofilm formation (Recht and Kolter, 2001; Bardouniotis et al., 2003) in 7H9 liquid with 10% glucose was carried out in six-well plates with or without glutamine. For rifampicin treatment, *in vitro* biofilms were grown in triplicate for 48 h on 96-well plates as described (Lebeaux et al., 2014) with minor modification. The planktonic bacteria were removed by washing with 1× PBS and biofilms were treated for 24 h with increasing concentrations of rifampicin (48.6–145.8 μM) with or without glutamine in M9 medium. There is no glucose in M9 medium while the control wells without antibiotics.

### Metabolome Assay and Data Analysis

*Mycobacterium smegmatis* was cultured in 250 mL bottles under starvation conditions, each sample with three repeats. For antibiotic treated samples, three 50 mL cultures were pelleted by centrifugation and washed twice with 1× PBS, followed by resuspending in M9 media without glucose, in the presence of rifampicin (145.8 μM), incubated 12 h with or without glutamine (Duan et al., 2016). For metabolome analysis, samples were pelleted and subjected to a μHPLC (1290 Infinity LC, Agilent Technologies) (Ivanisevic et al., 2013) coupled to a quadrupole time-of-flight (AB Sciex TripleTOF 6600) analysis. The raw MS data were converted into the MzXML files by using a ProteoWizard MSConvert tool and processed using the XCMS (Benton et al., 2008) for feature detection, retention time correction, and alignment. The metabolites were identified by accuracy mass (<25 ppm) and MS/MS data, which matched with our standard database. For multivariate statistical analysis, the web-based MetaboAnalyst^1^ system was used, followed by the Pareto scaling, principal component analysis (PCA) and partial least-squares-discriminant analysis (PLS-DA). Then leave one out cross-validation and response the permutation testing were used to evaluate the robustness of the model. The significantly different metabolites were determined based on the combination of a statistically significant threshold of variable influence of projection (VIP) value obtained from PLS-DA model and two-tailed Student’s *t-*test (*P-*value) on the raw data, and the metabolites with VIP values larger than 1.0 and *P*-values less than 0.05 were considered as significant.

### Transcriptome Assay and Data Analysis

The *M. smegmatis* was cultured in 250 mL bottles under starvation conditions, each sample with three repeats. For antibiotic treated samples, three 50 mL cultures were pelleted by centrifugation, washed twice with 1× PBS followed by resuspending in M9 medium destitute of glucose, treated with 145.8 μM rifampicin for 12 h in the presence/absence of glutamine (Duan et al., 2016). The procedure was adapted from reference (Mortazavi et al., 2008) with slight modification. Total RNA was isolated using the RNeasy mini kit (Qiagen, Germany). Paired-end libraries were synthesized by using the TruSeq RNA Sample Preparation Kit (Illumina, United States) according to TruSeq RNA Sample Preparation Guide. The cleaved RNA fragments were copied into first strand cDNA using reverse transcriptase and random primers. The cDNA fragments were synthesized afterward through an end repair process followed by the addition of a single “A” base, and then the fragments were ligated to the adapters. The products were then purified and enriched with PCR to create the final cDNA library. The cluster was generated by cBot with the library being diluted to 10 pM and then was sequenced on the Illumina HiSeq 2500 (Illumina, United States). The library construction and sequencing were performed at the Shanghai Biotechnology Corporation. The sequencing raw reads were preprocessed by filtering out the rRNA reads, sequencing adapters, short-fragment reads, and other low-quality reads. After genome mapping, the Cufflinks v2.1.1 was run with a reference annotation to generate a FPKM values for known gene models. Differentially, expressed genes were identified using Cuffdiff. The *P*-value significance threshold in multiple tests was set by the false discovery rate (FDR). The fold-changes were also estimated according to the FPKM in each sample. The expressed genes were selected by using the following filter criteria: FDR ≤ 0.05 and fold-change ≥ 2. The data can be found in GEO website with accession number GSE108001.

### NADH Measurement

*Mycobacterium smegmatis* cultures undergone nutrition starvation for 48 h were pelleted by centrifuged at 8,000 rpm (7990 *g*), 10 min and resuspended in M9 medium deprived of glucose, followed by exposing in 145.8 μM rifampicin with or without 2 mM glutamine for 12 h. The culture was harvested and diluted to OD_600_ = 0.2. For NADH measurement, cell pellets were washed twice with 1× PBS and resuspended with NADH extraction buffer (0.1 M HCl). Heat extraction was performed at 60°C for 5 min. NAD^+^ extraction buffer (0.1 M NaOH) were added to neutralize the extractions. Cells were vortexed and spun down at 12,000 rpm (13,800 g) for 5 min. The supernatant was collected for the EnzyChrom NAD^+^/NADH Assay Kit (BioAssay Systems). Then 40 μL of sample was transferred into each well of a clean, flat-bottom 96-well plate. For each reaction well, working reagents consisting of 60 μL assay buffer, 1 μL enzyme A, 1 μL enzyme B, 14 μL lactate, and 14 μL MTT were then added. The optical density (OD_565_) for time “zero” at 565 nm (520–600 nm) and OD_565_ after 15 min incubation at room temperature were recorded.

### The Reactive Oxygen Species Analyses

*Mycobacterium smegmatis* cultures undergone starvation for 48 h were pelleted by centrifugation at 8000 rpm (7,990 *g*), 10 min and resuspended in M9 medium without glucose, resuspended samples were exposed to 145.8 μM rifampicin for 24–72 h with or without 2 mM glutamine. The control cultures received no drug. For ROS analysis, aliquots were taken at indicated time points and diluted to OD_600_ = 0.2. The samples were treated by the Reactive Oxygen Species Assay Kit (Beyotime). DCFH-DA was diluted 1:1000 with 1× PBS. Pelleted samples were resuspended with diluted DCFH-DA at 37°C for 1 h. The harvested cultures were pelleted by centrifugation at 12,000 rpm (13,800 *g*) for 1 min and then washed twice with 1× PBS. Fluorescence was analyzed on a BD FACS Calibur (BD Biosciences) as previously described (Vilchèze et al., 2013).

### DNA Damages Analyses

*Mycobacterium smegmatis* cultures undergone nutrition starvation for 48 h were pelleted by centrifugation at 8,000 rpm (7,990 *g*) for 10 min and then resuspended with M9 medium without glucose. Resuspended samples were exposed to 145.8 μM rifampicin for 24–72 h with or without 2 mM glutamine. There is no antibiotics in the control cultures. The aliquots used for TUNEL analysis were removed at indicated time points, and treated with a TUNEL reaction mix from the *In situ* Cell Death Detection Kit (Roche Molecular Biochemicals. The samples were washed twice with 1× PBS and fixation solution was added (final concentration 2% PFA) at 25°C for 60 min. The culture was centrifuged and the fixative was removed by suction. Resuspended cells were soaked in penetrant for 2 min on ice. The inducer IPTG was added prior to fluorescence intensity measure and the samples were analyzed by flow cytometry as described (Vilchèze et al., 2013).

### ATP Measurement

*Mycobacterium smegmatis* cultures undergone nutrition starvation for 48 h were pelleted centrifugation at 8,000 rpm (7,990 *g*) for 10 min and then resuspended with M9 medium without glucose. Resuspended samples were cultured with or without 2 mM glutamine for 24–48 h. The ATP level was measured by using a BacTiter Glo kit (Promega) according to the instructions of manufacture. Fifty microliter Bactiter-Glo Reagent was added to 50 μL culture media. The contents were briefly mixed by shaking and incubated for 5 min. The background ATP was subtracted by using media alone. The luminescence was record with a fluorescence microplate reader.

## Results

### Synergism of Glutamine With Rifampicin Against Starving *M. smegmatis*

**FIGURE 1:**
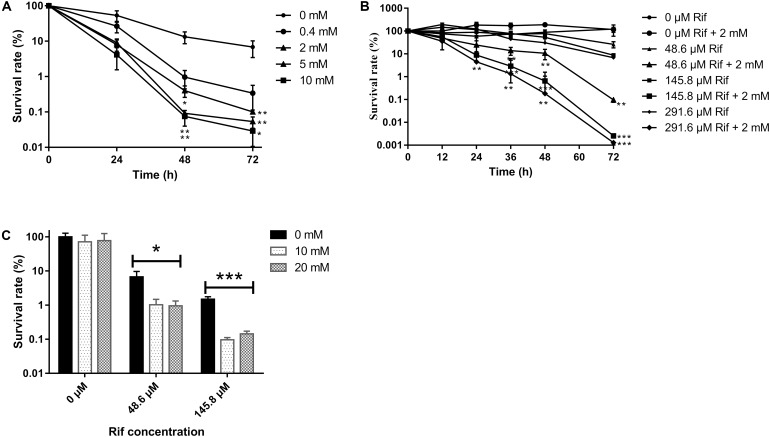
Survival of *Mycobacterium smegmatis* upon rifampicin exposure. **(A)** Starved wild type strain was exposed to rifampicin (145.8 μM) with an increasing concentration of glutamine (0–10 mM). **(B)** Starved wild type strain treated with an increasing concentration (0–291.6 μM) of rifampicin with presence or absence of 2 mM glutamine. **(C)** Biofilm bacteria treated with rifampicin (0–145.8 μM) and 0–20 μM glutamine. Aliquots were taken at the indicated times and were serially diluted and plated to determine CFUs. Values were compared with the control (without glutamine) at the same antibiotic concentration. Data are shown as mean ± SD of triplicate wells. Similar results were obtained in three independent experiments. Statistical analysis was performed using GraphPad Prism 6.0. The results were compared by Two-sided Student’s *t*-test. Differences were considered statistically significant with ^∗^*P* < 0.05, ^∗∗^*P* < 0.01, and ^∗∗∗^*P* < 0.001. Error bars represent standard deviation of the mean.

To test whether glutamine can alter the *M. smegmatis* persister sensitivity toward the antibiotic, the survival rate of planktonic *M. smegmatis* exposed to rifampicin (145.8 μM) in the presence/absence of glutamine (0.5–10 mM) was compared. The result showed that glutamine combined with rifampicin inhibited bacterial growth, and this effect was dose-dependent, with a significant inhibition at a range of 2–10 mM (the survival rate after 72 h was reduced 68-, 136-, and 235.52- fold at 2, 5, and 10 mM) (**Figure 1A**). To determine the bactericidal potency against the starved strains, rifampicin at concentrations ranged from 48.6 to 291.6 μM was added to the starved cultures, which produced survival rate at 4.68-, 14.02-, and 17.89- fold corresponding to rifampicin concentrations at 48.6, 145.8, and 291.6 μM, respectively. The results showed that glutamine can boost the efficacy of rifampicin against *M. smegmatis* in a dose-dependent way (**Figure 1B**).

### Glutamine Can Synergize With Rifampicin to Kill the Bacteria Within Biofilm

To assess whether glutamine can alter the sensitivity of *M. smegmatis* within the biofilm against antibiotics, the biofilm was subjected to rifampicin with or without glutamine. As expected, the survival rate of *Mycobacteria* within biofilm was reduce to 10 and 1% upon exposure to rifampicin at 48.6 and 145.8 μM (**Figure 1C**).

### Glutamine Enhanced Rifampicin Bactericidal Effect Was Irrelevant to Increased Nitrogen Source Availability

**FIGURE 2:**
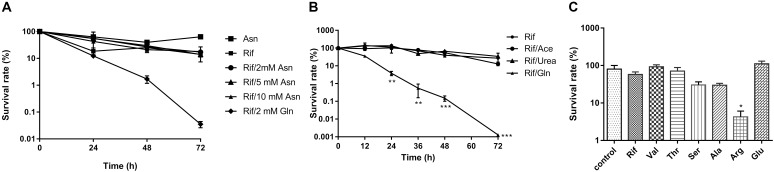
The effect of amino acids and nitrogen sources on synergism of rifampicin. **(A)** WT strain treated with rifampicin alone after starvation or in combination with 2 mM asparagine or 2 mM glutamate for 0–72 h. **(B)** WT strains with rifampicin alone after starvation or in combination with 2 mM acetamide, urea, or glutamine for 72 h. **(C)** WT strain treated with rifampicin alone after starvation or in combination with 2 mM valine, threonine, serine, alanine, arginine, or glutamate for 72 h. Aliquots were taken at the indicated times and plated to determine CFUs. Values were compared with the control (without amino acid) at the same antibiotic concentration. Data were shown as mean ± SD of triplicate wells. Similar results were obtained in three independent experiments. Statistical analysis was performed using GraphPad Prism 6.0. The results were compared by Two-sided Student’s *t*-test. Differences were considered statistically significant with ^∗^*P* < 0.05, ^∗∗^*P* < 0.01, and ^∗∗∗^*P* < 0.001. Error bars represent standard deviation of the mean. Rif, rifampicin; Val, valine; Thr, threonine; Ser, serine; Ala, alanine; Arg, arginine; Glu, glutamine; ace; acetamide; gln, glutamine.

To investigate whether glutamine *enhanced* rifampicin efficacy was due to its role as nitrogen source, we treated the starved cells with various amino acids, such as valine, threonine, serine, alanine, arginine, glutamate, asparagine, and glutamine. We found that asparagine, threonine, glutamate, and valine failed to synergize the rifampicin activity (**Figure 2A**), though that glutamate is the deaminated product of glutamine (**Figure 2C**). However, arginine (14.45- fold) (**Figure 2C**), alanine or serine can slightly enhance rifampicin efficacy. To further explore the effect of nitrogen source on rifampicin efficacy, acetamide and urea were used to treat cells in combination with rifampicin. Both agents failed to enhance the rifampicin activity against *M. smegmatis* (**Figure 2B**). Thus, the synergistic effect on rifampicin is specific with glutamine.

### The Combination of Rifampicin and Glutamine Accelerated the Degradation of Amino Acid and TCA Cycle

**FIGURE 3:**
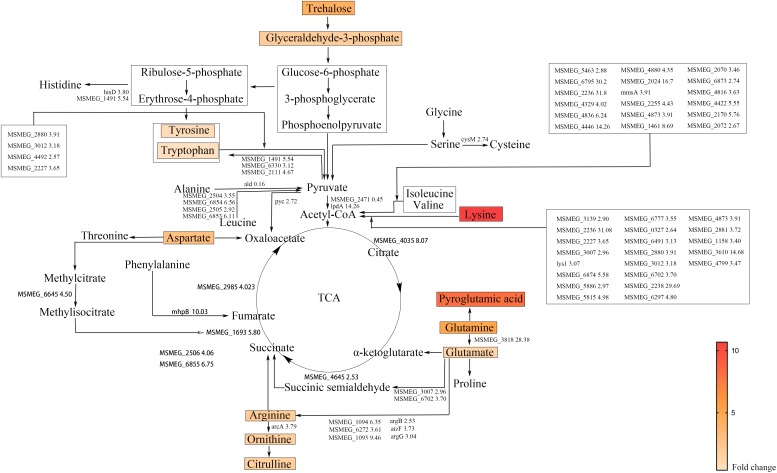
Amino acid degradation pathways and TCA cycle in *M. smegmatis*. Metabolites identified by metabolome are marked by colored box and names. Colors represent the fold change of metabolites level. The enzymatic reactions and related genes identified by transcriptome are indicated by black lines and gene name followed with fold change. The gene names were taken from the *M. smegmatis* genome database (http://mycobrowser.epfl.ch/smegmalist.html).

To provide mechanistic insights into the synergistic effect of glutamine on rifampicin, the metabolome difference upon glutamine addition was explored. The contents of 10 amino acids were dramatically changed by the addition of glutamine (**Figure 3**), namely lysine (10.68- fold), pyroglutamic acid (8.12- fold), glutamine (5.07- fold), aspartate (2.94- fold), ornithine (1.69- fold), citrulline (1.72- fold), glutamate (1.33- fold), and arginine (2.01- fold). The most significantly changed amino acids were the degraded products of cell nutrients. **Figure 3** showed the change of key genes involved in amino acids metabolism, such as *lys1* (3.07- fold) underlying the lysine conversion into citrate via condensing with an oxaloacetate and acetyl-CoA, *glsA* (*MSMEG_3818*) (28.38- fold) responsible for the transformation of glutamine to glutamate as well as arginine, *mhpB* (10.03- fold) catalyzing the phenylalanine degradation into fumarate, methylcitrate dehydratase (*MSMEG_6645*) (4.50- fold), carboxyvinyl-carboxyphosphonate phosphorylmutase (*MSMEG_2506*) (4.06- fold) and *MSMEG_6855* (6.75- fold) underlying the succinate formation from the asparate degradation.

### Reactive Oxygen Species Level Correlates the Synergistic Action of Glutamine With Rifampicin

To investigate whether glutamine addition can accelerate the TCA cycle, the intracellular ATP level was measured by fluorescence microplate reader with the starved cultures in the presence/absence of 2 mM of glutamine, glutamate, or arginine. Intracellular ATP level increased 2.44- fold (**Figure 5A**) at 72 h after glutamine was added. However, the addition of glutamate or arginine caused little increase in the intracellular ATP content (**Figure 5A**). To explore the effect of glutamine on the NAD^+^/NADH ratio of bacteria treated with rifampicin/glutamine, we monitored NAD^+^ and NADH concentrations in *M. smegmatis* treated with rifampicin alone or in combination with glutamine. The rifampicin exposure can increase NAD^+^/NADH ratio, while rifampicin–glutamine combination can lower the NAD^+^/NADH ratio (**Figure 5B**, left panel). The change fold of NAD^+^/NADH ratio from 24 to 48 h was determined (**Figure 5B**, right panel). As expected, rifampicin and glutamine exposure can increase ROS level (**Figure 5C**). The increased DNA damage also corroborated the higher level of ROS. The combination of rifampicin and glutamine resulted in more *M. smegmatis* DNA damage han rifampicin alone, as revealed by the fluorescent probe specific to the DNA damage. The DNA damage level increased at 72 h (**Figure 5D**). The combination of rifampicin and cysteine showed negligible synergy against the starved culture (Supplementary Figure 1). Taken together, the results implicated that glutamine might synergize the efficacy of rifampicin against *M. smegmatis* by increasing the production of endogenous ROS.

## Discussion

Tuberculosis, caused by *M. tuberculosis*, remains a major public health concern (WHO, 2016). Persistence and antibiotic resistance of the pathogen represent two main obstacles for TB eradication. Persisters play a significant role in the antibiotic tolerance (Keren et al., 2004). Mechanistic insights into persistence can find better targets for novel drugs to shorten the tuberculosis treatment duration. In this study, we found that the glutamine can sensitize *M. smegmatis* to rifampicin-mediated killing (**Figures 1A,B**) (di Mauro et al., 1969; Quenelle et al., 1999; Campbell et al., 2001), consistent with previous publications showing that high concentration arginine can synergize with gentamicin against *E. coli* (Recht and Kolter, 2001; Lebeaux et al., 2014; Shan et al., 2015). Notably, prolonged starvation can enhance the tolerance to rifampicin though the addition of glutamine increased the activity of rifampicin (Supplementary Figure 2). The glutamine synergistic effect is obvious for both planktonic bacteria and the bacteria within biofilm (**Figure 1C**). Some other amino acids showed similar effect. Arginine synergized with gentamicin against *E. coli*. The glutamine alone failed to show sterilization effect (**Figure 1C**), consistent with previous report on the biofilm development (Recht and Kolter, 2001) (Supplementary Figure 3). Taken together, these results showed that glutamine can synergize with rifampicin against *M. smegmatis* within the biofilm.

Since, the TCA cycle is essential for the production of energy and reactive oxygen species, we speculated that the addition of glutamine might accelerate the TCA cycle. The transcriptome data (**Figure 3**) showed that glutamine starvation differentially regulated many genes involved in amino acid metabolism, especially the up-regulation of genes involved in degradation of amino acid (Wilmes et al., 2011; Lechner et al., 2014). The degraded amino acids can fuel the TCA cycle. Herein, the rifampicin–glutamine combination can accumulate more ROS, which rendered more DNA damages. The addition of glutamine might increase the metabolic rate of persisters, as evidenced by both transcriptome and metabolome data. The persisting bacteria might be reactivated, therefore more susceptible to antibiotics targeting the active cellular processes.

**FIGURE 4:**
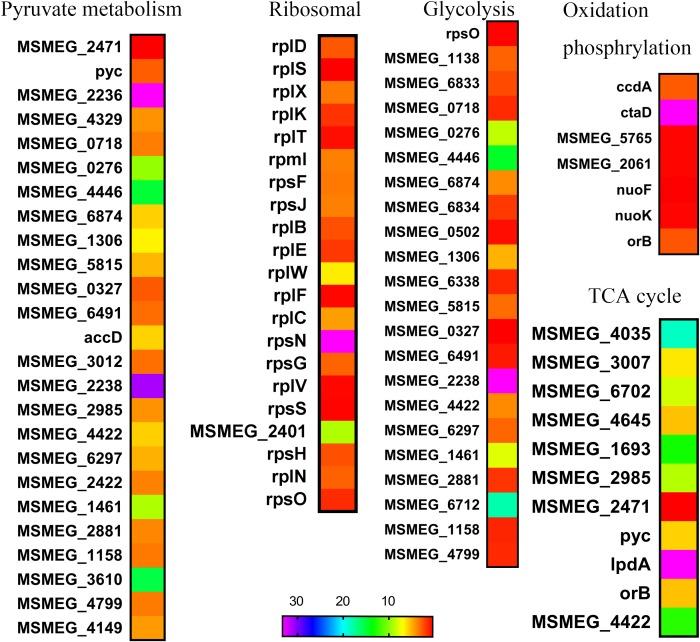
Heat maps of central metabolism pathways. The relative fold change of expression level in several biosynthetic pathways was calculated and visualized over time using Excel, heat map was made with GraphPad Prism 6.0.

We found that glutamine synergize with rifampicin against the starved *M. smegmatis*. In fact, amino acids such as asparagine (**Figure 2A**), glutamate, alanine, serine, valine, and threonine failed to show such synergistic effect (**Figure 2C**). The arginine combined with gentamicin altering the pH value (Lebeaux et al., 2014), unlike we noted that only moderately boosted rifampicin activity (**Figure 2C**). The hydrolysis of asparagine can promote the acid stress resistance, asparagine transporter AnsP2 and the secreted asparaginase AnsA are also involved (Gouzy et al., 2014a). The synergistic effect of glutamine on rifampicin against *Mycobacteria* might be indirectly mediated by the central metabolism (Lewis, 2010), like other amino acids (Shan et al., 2015; Vilcheze et al., 2017). The glutamine starvation represents a good window to understand the changed central metabolism, respiration and the expression of stress-responsive genes (Ye et al., 2009; Jessberger et al., 2013). The data are consistent with the previous reports (Ye et al., 2009; Keren et al., 2011) that the addition of glutamine accelerated both the central metabolism and TCA cycle (**Table 1**), fueled by the influx of degraded amino acids. The central metabolism acceleration (Rhee et al., 2011) was also evidenced by the up-regulated transcription of genes involved in pyruvate metabolism (Keren et al., 2011; Rhee et al., 2011), glycolysis metabolism (Keren et al., 2011; Rhee et al., 2011) in the transcriptome data (**Figure 4**). The ribosomal constituents, as well as components of respiration chain down-regulated in the dormancy model (Betts et al., 2002), were upregulated in rifampicin–glutamine treated samples (**Figure 4**). The conversion of glutamine into amino acids such as lysine, arginine, and tryptophan, and subsequent up-regulation of TCA cycle might underlie the synergistic effect of glutamine. However, since transcriptome profile experiment compared glutamine with buffer alone and not another irrelevant nitrogen source, it is not clear whether the expression patterns represent changes that are specific to glutamine supplementation or just nitrogen supplementation. Whether glutamine can function as a signaling molecule to regulate gene expression in persisters as in plants (Kan et al., 2015) remains to be determined.

**Table 1:** Genes involved in the TCA cycle showing altered expression.

Gene number	Functions	Fold change	Up/down
MSMEG_4035	Citrate synthase	8.07	Up
MSMEG_3007	Succinate-semialdehyde dehydrogenase	2.96	Up
MSMEG_6702	[NADP+] Succinate-semialdehyde dehydrogenase	3.70	Up
MSMEG_4645	2-Oxoglutarate ferredoxin oxidoreductase subunit beta	2.53	Up
MSMEG_1693	Succinate dehydrogenase	5.80	Up
MSMEG_2985	Fumarate hydratase class I	4.02	Up
MSMEG_2471	Pyruvate dehydrogenase subunit alpha	0.45	Down
pyc	Pyruvate carboxylase	2.72	Up
lpdA	Dihydrolipoamide dehydrogenase	14.26	Up
orB	2-Oxoglutarate ferredoxin oxidoreductase subunit beta	2.53	Up
MSMEG_4422	Oxidoreductase	5.55	Up

**FIGURE 5:**
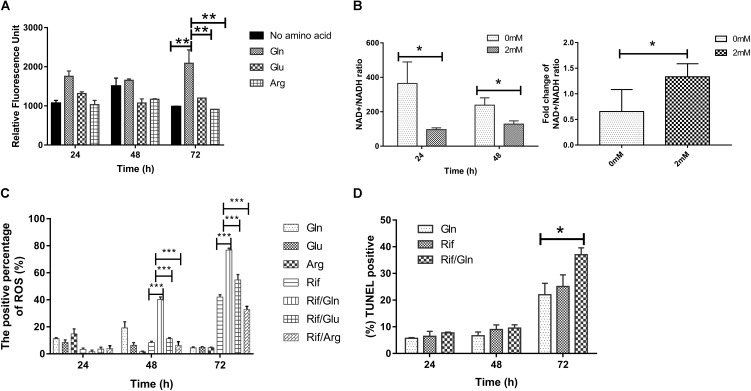
The effect of rifampicin and exogenous glutamine on the production of reactive oxygen species (ROS). **(A)** Cultures of nutrition starvation were treated with or without 2 mM glutamine, glutamate or arginine by 24–72 h before measuring the ATP level by GFP fluorescence. **(B)** The ratio of NAD+/NADH exposed to rifampicin (24 and 48 h) in presence or absence of 2 mM glutamine. **(C)** Percentages of intracellular increase in ROS generated in the presence of 2 mM glutamine, glutamate, or arginine compared with treatment with rifampicin alone. **(D)** Percentages of intracellular DNA damage generated in the presence of 2 mM glutamine compared with treatment with rifampicin alone. Values were compared with the control (without glutamine) at the same antibiotic concentration. Data are shown as mean ± SD of triplicate wells. Similar results were obtained in three independent experiments. Statistical analysis was performed using GraphPad Prism 6.0. The results were compared by Two-sided Student’s *t*-test. Differences were considered statistically significant with ^∗^*P* < 0.05, ^∗∗^*P* < 0.01, ^∗∗∗^*P* < 0.001. Error bars represent standard deviation of the mean. Gln, glutamine; Rif, rifampicin; glu, glutamate; arg, arginine.

Rifampicin, which binds to the beta subunit of the RNA polymerase encoded by *rpoB*, functions by inhibiting DNA-dependent RNA polymerase. Few reports concerned the involvement of OH formation in rifampicin action (Piccaro et al., 2014). The combination of glutamine and rifampicin can enhance the rifampicin efficacy against *M. smegmatis* by increasing the production of endogenous ROS, but glutamate and arginine failed to show the same effect (**Figure 5C**). This effect was also observed for well-established superoxide-derived oxidation of iron–sulfur clusters, which in turn stimulate the Fenton reaction-mediated generation of highly destructive hydroxyl radicals (Dwyer et al., 2007). The reactive oxygen species is largely from the product of oxygen during the electron transport along the respiratory chain and the conversion of NADH into NAD^+^ (Imlay and Fridovich, 1991a,b). The decreased NAD^+^/NADH ratio (**Figure 5B**) can tip the balance of intracellular redox. The rifampicin/glutamine caused more cell death as evidenced by increased DNA damage (**Figure 5D**). Thus the data suggested that glutamine can synergize with rifampicin by promoting the intracellular ROS production. This synergism might be used to combat multidrug resistant bacteria and eradicate persisters. The metabolome data revealed an increase of the intracellular amino acids and decrease of protective trehalose (Jessberger et al., 2013) (**Figure 3**). Genes involved in fatty acid biosynthesis were upregulated in the transcriptome data. Ribosomal proteins were up-regulated in strains exposed to rifampicin and glutamine (**Figure 4**). Data also showed that genes involved in respiration, glycolysis, and oxidative phosphorylation were up-regulated (**Figure 4**), in contrast to previous reports about persisters (Shah et al., 2006; Keren et al., 2011). These changes of transcriptome were confirmed by the increase of ATP and ROS (**Figures 5A,C**). The increased intracellular ATP in *M. smegmatis* treated with rifampicin–glutamine combination might also play a role in the decrease of persister tolerance (**Figure 5A**). ATP is required for the action of most antibiotics, and the decrease of ATP might increase the drug tolerance (Shan et al., 2017). A good example about ATP is that, once a culture stops growing, peptidoglycan synthesis ceases, and cells became tolerant to antibiotics targeting cell wall (Tuomanen et al., 1986). The increase of intracellular ATP level signals a disruption of *M*. *smegmatis* persistence and parallels with accelerated TCA cycle.

Many details underlying the increase of ROS remain to be explored. The exogenous glutamine can decrease the rate of mistranslation (Javid et al., 2014). It is interesting to know whether the changed glutamine/glutamate ratio is elicited by altered mistranslation, which is facilitated by glutamyl-tRNA (Su et al., 2016). The transcription of glutamyl-tRNA encoding gene was up-regulated (Supplementary Table 1). The addition of glutamate failed to produce the effect of glutamine addition (Supplementary Figure 4). ROS and energy metabolism disruption are involved in the synergistic effect of glutamine with rifampicin. The study showed that glutamine might be included as an adjuvant to synergize with rifampicin to improve the control of *M. tuberculosis* persisters.

## Author Contributions

XH, XD, JL, JN, SiY, and JuX performed the experiments. XH, XD, and JL analyzed the data. XH, XD, and JuX designed the study and wrote the paper. All authors have read and approved the manuscript.

## Conflict of Interest Statement

The authors declare that the research was conducted in the absence of any commercial or financial relationships that could be construed as a potential conflict of interest.

## Footnotes

1 www.metaboanalyst.ca
