# Supplementary material for: The Synergistic Effect of Exogenous Glutamine and Rifampicin Against Mycobacterium Persisters
Source: Front Microbiol. 2018 Jul 20;9:1625. doi: 10.3389/fmicb.2018.01625 (PMC6062616; doi:10.3389/fmicb.2018.01625)
Supplement: Supplementary file 1 [file Image_1.PDF]

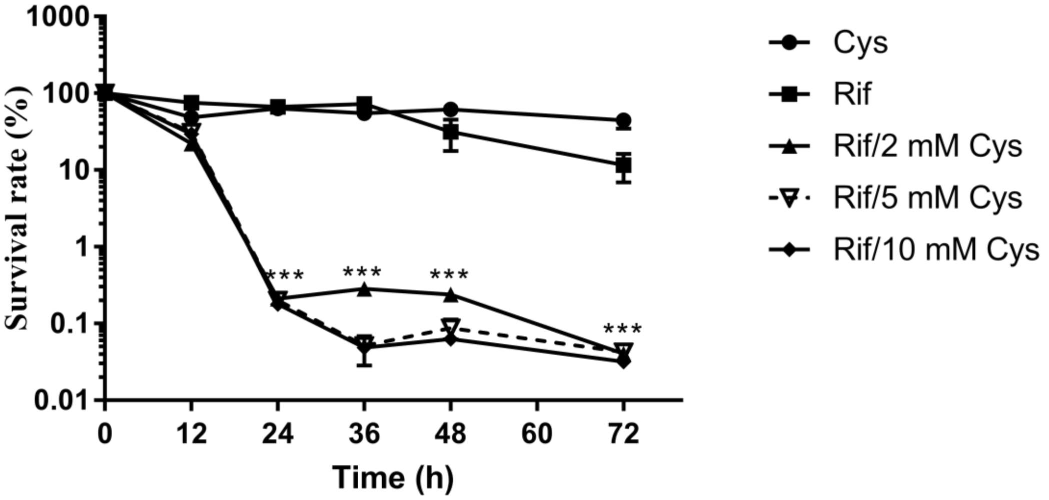

Supplement Figure 1. Cysteine synergizes with rifampicin against starved *M. smegmatis* strains. Starved strains treated with rifampicin and cysteine (2mM, 5mM, 10mM) for 0h-72h. Aliquots were taken atthe indicated times and plated to determine CFUs. The data are shown as means ± SD of triplicate wells. The similar results were obtained in three independent experiments. Values were compared with the control (without cysteine). Statistical analysis was performed using GraphPad Prism 6.0. The results were compared by Student's *t* test. Differences were considered statistically significant with ***P< 0.001. Error bars represent standard deviation of the mean. Rif, rifampicin; Cys, cysteine.
