# Supplementary material for: The Synergistic Effect of Exogenous Glutamine and Rifampicin Against Mycobacterium Persisters
Source: Front Microbiol. 2018 Jul 20;9:1625. doi: 10.3389/fmicb.2018.01625 (PMC6062616; doi:10.3389/fmicb.2018.01625)
Supplement: Supplementary file 2 [file Image_2.PDF]

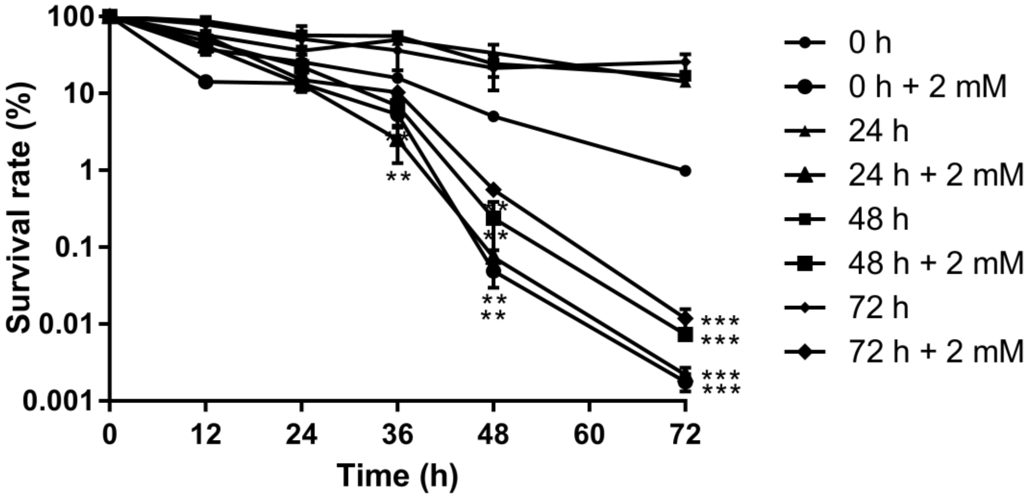

Supplement Figure 2. Survival *M. smegmatis* under rifampicin exposure with increasing nutrition starvation. The wild type (WT) strain were treated with or without glutamine under rifampicin. Aliquots were taken atthe indicated times and plated to determine CFUs. The data are shown as means ± SD of triplicate wells. The similar results were obtained in three independent experiments. Values were compared with the control (without glutamine) at the same antibiotic concentration. Statistical analysis was performed using GraphPad Prism 6.0. The results were compared by Student’s *t* test. Differences were considered statistically significant with $^{**}P < 0.01$, $^{***}P < 0.001$. Error bars represent standard deviation of the mean. 24h,48h,72h: starved with 24h; 48h,72h.
