# Supplementary material for: The Synergistic Effect of Exogenous Glutamine and Rifampicin Against Mycobacterium Persisters
Source: Front Microbiol. 2018 Jul 20;9:1625. doi: 10.3389/fmicb.2018.01625 (PMC6062616; doi:10.3389/fmicb.2018.01625)
Supplement: Supplementary file 3 [file Image_3.PDF]

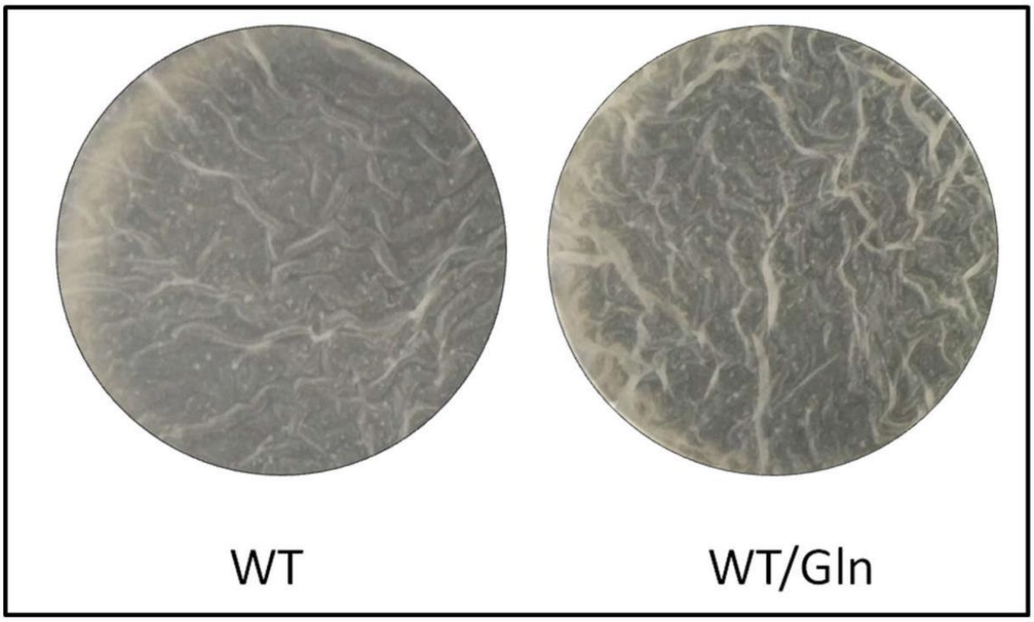

Supplementary Figure 3. Biofilm formation of *M. smegmatis* treated with or without 2mM glutamine. Log-phage cultures of *M. smegmatis* were diluted into 7H9 liquid with 3% glucose in 1:1000, biofilm was observed after 72h. WT: Wild type; WT/Gln: WT strains treated with glutamine.
