# Supplementary material for: The Synergistic Effect of Exogenous Glutamine and Rifampicin Against Mycobacterium Persisters
Source: Front Microbiol. 2018 Jul 20;9:1625. doi: 10.3389/fmicb.2018.01625 (PMC6062616; doi:10.3389/fmicb.2018.01625)
Supplement: Supplementary file 5 [file Table_1.PDF]

Supplementary Table 1. Genes involed in glutamyl-tRNA synthesis

Gene number	Functions	Fold change	UpDown
gatA	glutamyl-tRNA(Gln)/aspartyl-tRNA(Asn) amidotransferase subunit alpha	16.22	Up
MSMEG_0485	amidase	4.67	Up
MSMEG_1090	glutamyl-tRNA aminoacylation	2.66	Up
MSMEG_2986	amidohydrolase	3.04	Up
MSMEG_3970	glutamyl-tRNA(Gln) amidotransferase subunit A	3.17	Up
